# Supplementary material for: Functional alterations of the prefrontal circuit underlying cognitive aging in mice
Source: Nat Commun. 2023 Nov 9;14:7254. doi: 10.1038/s41467-023-43142-0 (PMC10636129; doi:10.1038/s41467-023-43142-0)
Supplement: Supplementary file 1 — Supplementary Information [file 41467_2023_43142_MOESM1_ESM.pdf]

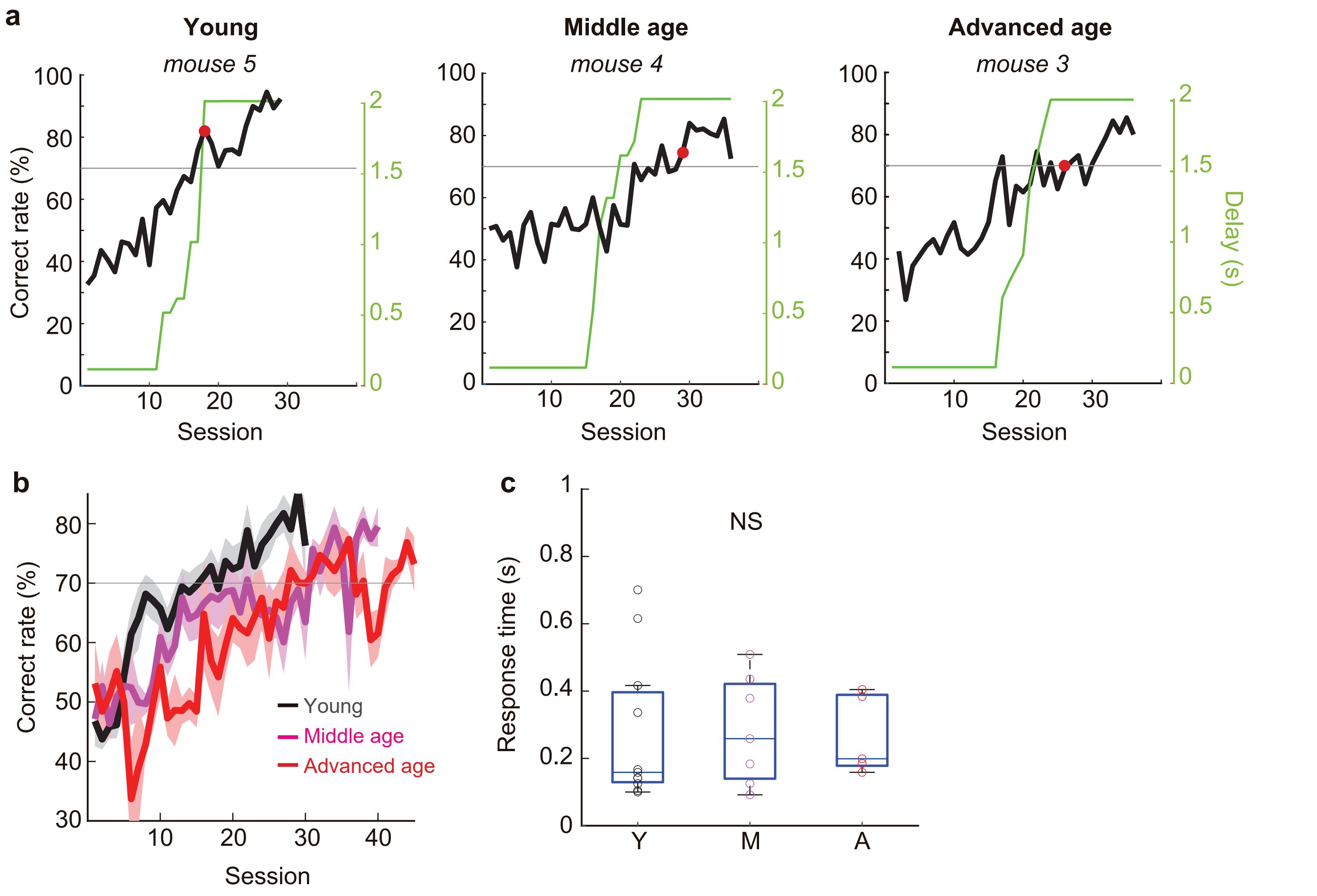

Supplementary Figure 1. Learning curve in the bimodal delayed 2-AFC task and response time during calcium imaging. a, Correct rate (black trace) and delay length (green trace) as a function of daily sessions for example individual mice in each age group Red dot, session in which the task performance reached the behavioral criteria (70% or higher correct rate for at least 2 consecutive sessions). **b**, Mean correct rate as a function of daily sessions averaged across all the mice (n = 11 young, n = 7 middle-aged, n = 5 advanced-aged mice). Colored shadings, ± s.e.m. **c**, Response time in imaging sessions did not differ among the age groups (U = 101, p = 0.79 for young vs. middle-aged, U = 85, p =0.38 for young vs. advanced-aged, U = 45, p = 0.99 for middle-aged vs. advanced-aged; Wilcoxon rank-sum test). Box plots show the median and the 25th and 75th percentiles as box edges and the whiskers extend to the 5th and 95th percentiles. Open circles, individual mice. Y, young; M, middle age; A, advanced age. Source data are provided as a Source Data file.

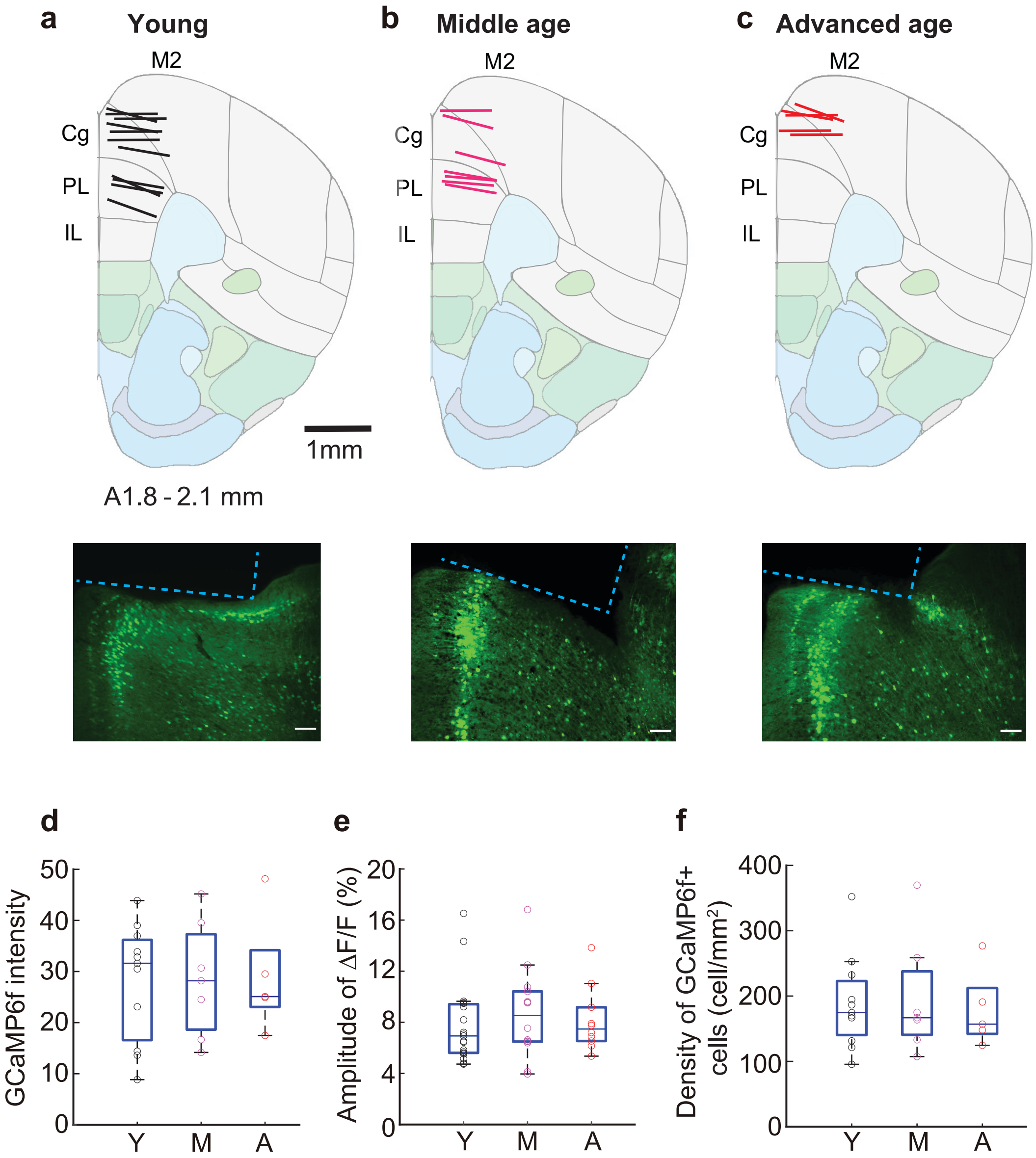

Supplementary Figure 2. Identification of calcium imaging sites and GCaMP6f expression. a-**c**, Top, GRIN lens implantation sites in all the CamKIIα-Cre mice from young (**a**), middle-aged (**b**), and advanced-aged mice (**c**), overlaid on the brain atlas modified from Allen Mouse Brain Coronal section (image 38), atlas.brain-map.org. Colored lines denote the tip of the implanted GRIN lens. Bottom, example images of GCaMP6f-expressing neurons from each age group. Light-blue dotted line, outline of GRIN lens. Scale bars, 100 μm. **d**, Fluorescent intensity of GCaMP6f in histology sections (n = 11 young mice, n = 7 middle-aged mice, n = 5 advance-aged mice). The mean fluorescent intensity of GCaMP6f was calculated as the mean pixel intensity of GCaMP6f-positive cells normalized by the mean background intensity outside the brain slice. **e**, Amplitude of DF/F of calcium events during the resting state. The maximum dF/F amplitude across all calcium events was calculated for each neuron, and then the median was computed across all the imaged neurons in each imaging session. **f**, Density of GCaMP6f-positive cells in histology sections of the imaged mice. Box plots show the median and the 25th and 75th percentiles as box edges and the whiskers extend to the 5th and 95th percentiles. Open circles, individual mice. Y, young; M, middle age; A, advanced age. Source data are provided as a Source Data file.

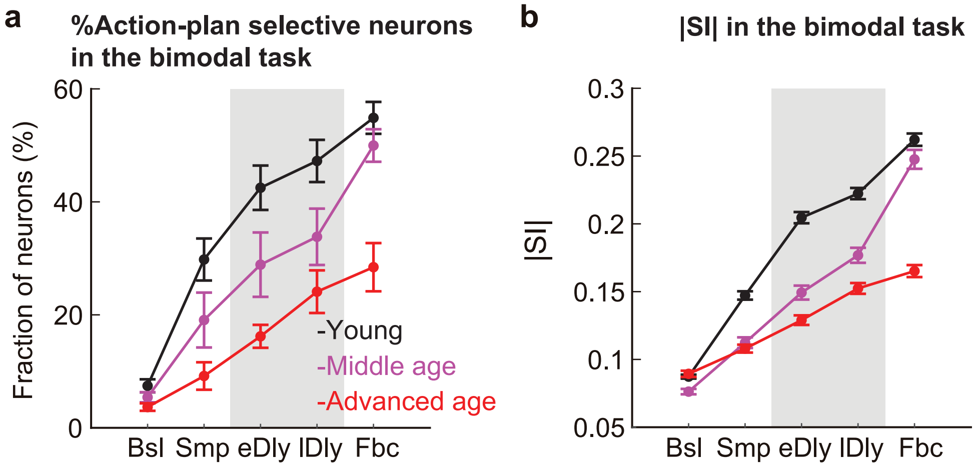

Supplementary Figure 3. Action-plan selective activity in each task period during the bimodal 2-AFC task. a, Mean fraction of the neurons with significant action-plan selective activity during each task period of the bimodal d2-AFC task averaged for all the imaging sessions (young, *n* = 22 sessions, 11 mice; middle age, *n* = 14 sessions, 7 mice; advanced age, *n* = 10 sessions, 5 mice). The fraction of action-plan selective neurons was significantly different among the age groups ($F(2,220)$ = 33.8, p < 0.0001, significant main effect of the age group with two-way ANOVA). **b,** Mean absolute selectivity index for action plan (|SI|) (young, *n* = 2,245 neurons; middle age, *n* = 859 neurons; advanced age, *n* = 968 neurons). The absolute selectivity index was significantly different among the age groups ($F(2,20345)$ = 238.1, p < 0.0001, significant main effect of the age group with two-way ANOVA). Gray shadings, delay period. Error bars, ± s.e.m. Bsl, baseline; Smp, sample; eDly, early delay; lDly, late delay; Fbc, feedback period. Source data are provided as a Source Data file.

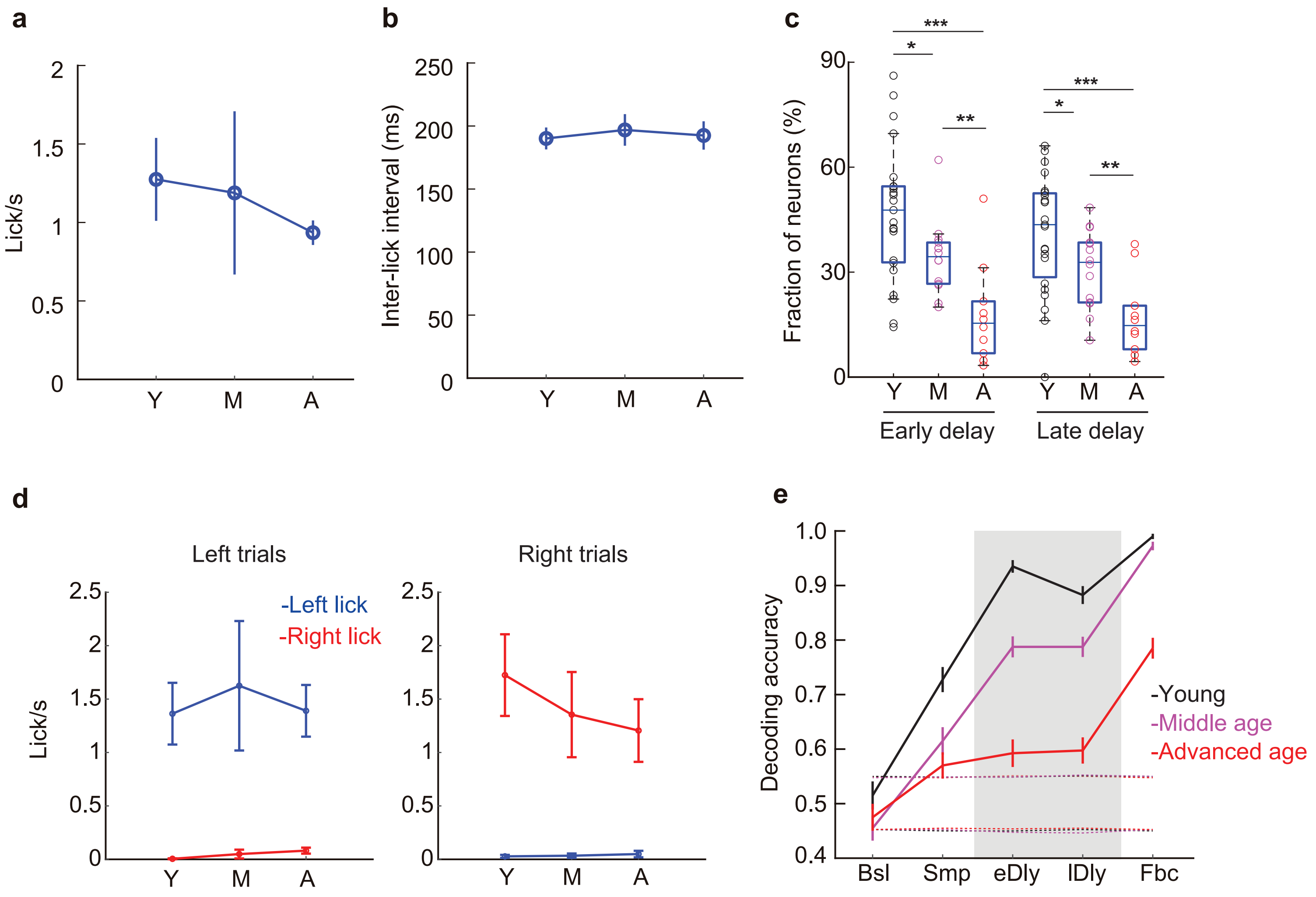

Supplementary Figure 4. Licking response and action-plan signal during the delay period in the bimodal d2-AFC task. a, Mean lick rate during the delay in the bimodal task averaged across mice (young, n = 11 mice; middle age, n = 7 mice; advanced age, n = 5 mice). The mean lick rate did not differ among the age groups ($F(2,20)$ = 0.23, p = 0.80, one-way ANOVA). **b,** Mean inter-lick interval (ILI) during the delay averaged across mice. The ILI did not differ among the age groups ($F(2,20)$ = 0.08, p = 0.92, one-way ANOVA). **c,** Fraction of neurons whose activity was significantly modulated by the early and late delay variables after regressing out the lick variables in GLM (generalized linear model, Methods) for all the imaging sessions (young, n = 23 sessions, 11 mice; middle age, n = 14 sessions, 7 mice; advanced age, n = 10 sessions, 5 mice). Box plots, the median and the 25th and 75th percentiles as box edges with the whiskers extending to the 5th and 95th percentiles. *U = 508, p = 0.027, **U = 225, $p = 3.7 \times 10^{-3}$, ***U = 481.5, $p = 4.2 \times 10^{-4}$ for early delay, *U = 506, p = 0.032, **U = 221, $p = 7.7 \times 10^{-3}$, ***U = 476, $p = 9.3 \times 10^{-4}$ for late delay, Wilcoxon rank-sum test. **d,** Mean left- and right-lick rate during the delay of left and right trials averaged across mice. **e,** Decoding accuracy of action plan using trials with no premature licks during the delay. Decoding accuracy during the early and late delay (gray shading) in the young group was higher than that in the other age groups (p < 0.001, two-way ANOVA (period x age group) with Bonferroni post hoc test). Error bars, ± s.e.m. across 100 iterations. The horizontal dotted lines, 97.5th and 2.5th percentiles of the shuffled data. Bsl, baseline; Smp, sample; eDly, early delay; lDly, late delay; Fbc, feedback period. Error bars, ± s.e.m. Y, young; M, middle age; A, advanced age. Source data are provided as a Source Data file.

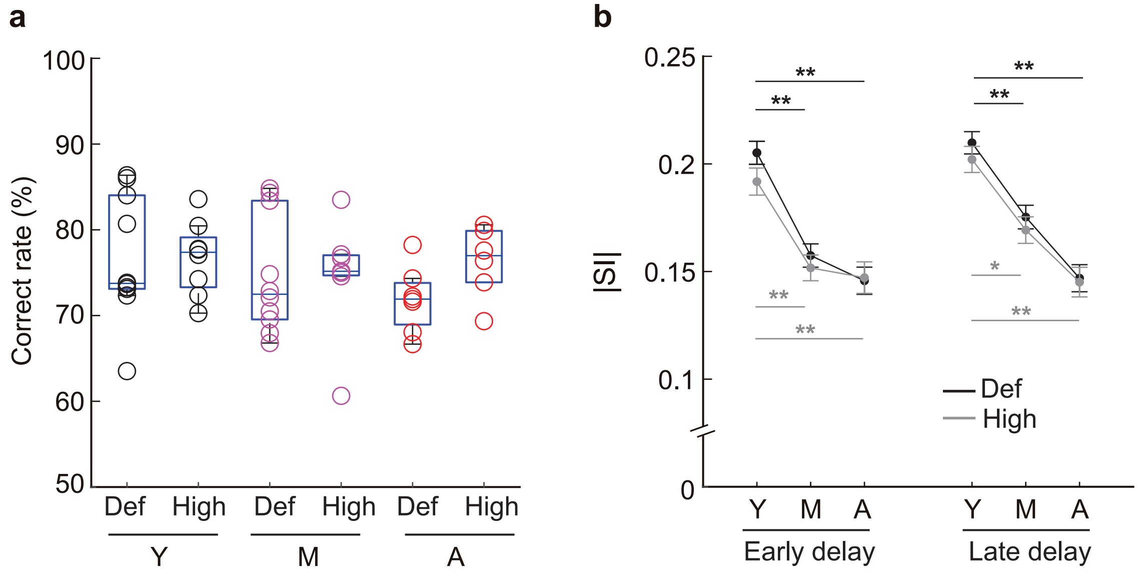

Supplementary Figure 5. Impact of reward size on behavior and action-plan selectivity. a, Correct rate in the tactile d2-AFC task with a variable reward size (young, *n* = 10 default and 8 high reward sessions, 5 mice; middle age, *n* = 10 default and 7 high reward sessions, 5 mice; advanced age, *n* = 7 default and 6 high reward sessions, 4 mice). Open circles, individual sessions. Def, default reward condition, High, high reward condition. Box plots show the median and the 25th and 75th percentiles as box edges and the whiskers extend to the 5th and 95th percentiles. Y, young; M, middle age; A, advanced age. **b**, Mean absolute selectivity index for action plan (|SI|) (young, *n* = 1,045 and 759 neurons; middle age, *n* = 659 and 529 neurons; advanced age, *n* = 407 and 376 neurons; for Def and High reward conditions, respectively). Error bars, ± s.e.m. **p* < 0.005, ***p* < 0.001, two-way ANOVA (reward size x age group) with Bonferroni post hoc test. Source data are provided as a Source Data file.

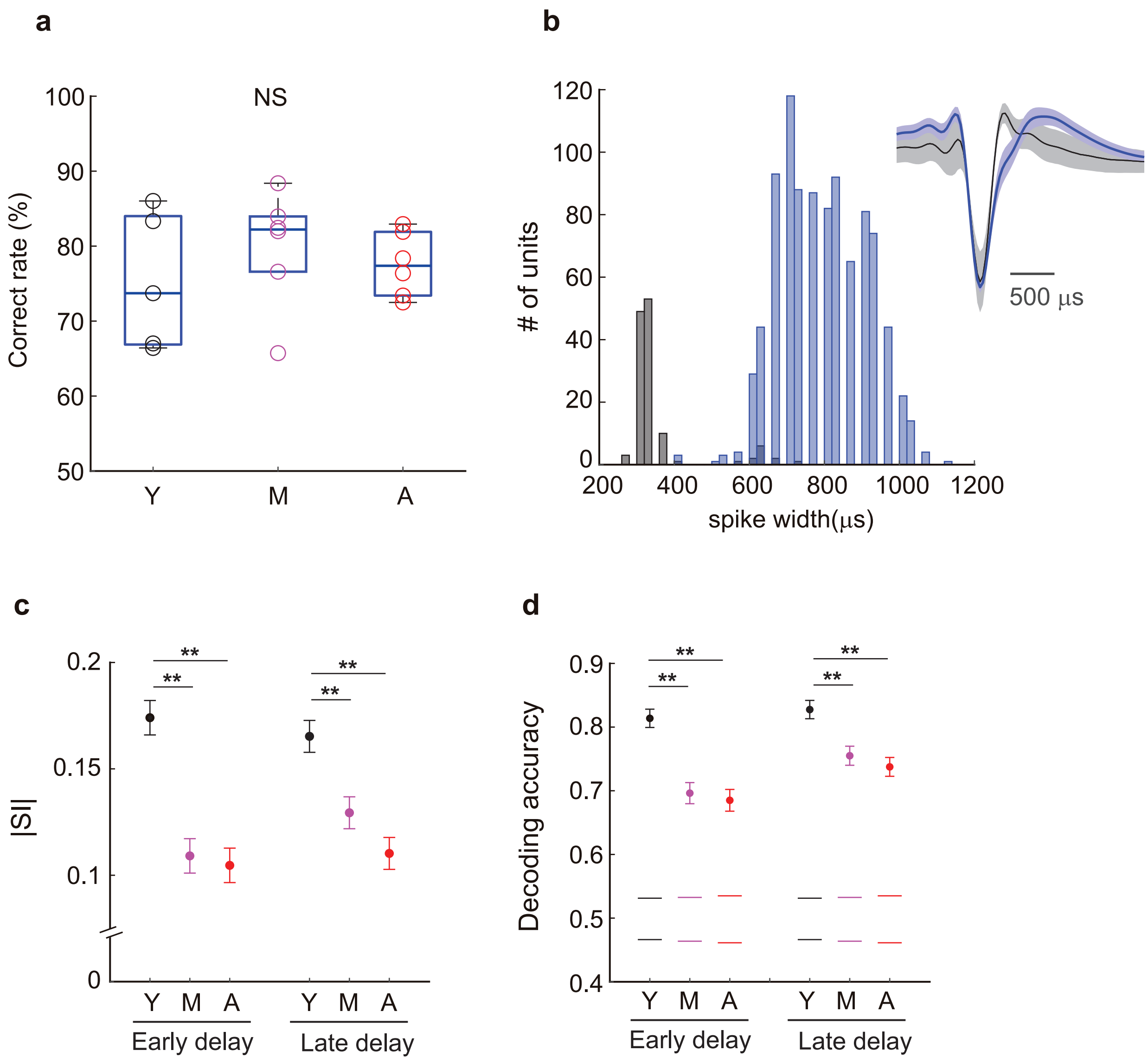

Supplementary Figure 6. Age-dependent decline in action-plan selectivity based on spiking activity. a, Correct rate during the electrophysiology recording sessions with the tactile d-2AFC task (young, n = 5 sessions, 2 mice; middle age, n = 6 sessions, 2 mice; advanced age, n = 6 sessions, 2 mice). Open circles, individual sessions. NS, no significant difference among age groups (U = 27, p = 0.67 for young vs. middle-aged, U = 29, p = 0.93 for young vs. advanced-aged, U = 46, p = 0.31 for middle-aged vs. advanced-aged; Wilcoxon rank-sum test). Box plots show the median and the 25th and 75th percentiles as box edges and the whiskers extend to the 5th and 95th percentiles. Y, young; M, middle age; A, advanced age. **b**, Distribution of spike width (trough-to-peak interval) of all recorded neurons. Blue and gray denote Broad-spiking (BS) and narrow-spiking (NS) neurons, respectively. Inset, the mean spike waveform for BS (blue) and NS (gray) neurons. The spike amplitude was normalized by its trough-peak amplitude for each neuron. Shadings, ± s.e.m. **c**, Mean absolute selectivity index for action plan (|SI|) (young, n = 225 neurons; middle age, n = 352 neurons; advanced age, n = 362 neurons). Error bars, ± s.e.m. **p < 0.001, one-way ANOVA with Bonferroni post hoc test. **d**, Decoding accuracy of action plan in each of the delay periods using 200 randomly selected neurons for each age group (Methods). Decoding accuracy during the early and late delay periods in the middle-aged and the advanced-aged groups was significantly lower than that in the young group (p < 0.001, one-way ANOVA with Bonferroni post hoc test). Error bars, ± s.e.m. across 100 iterations. Horizontal lines, 97.5th and 2.5th percentile of the shuffled distribution. Source data are provided as a Source Data file.

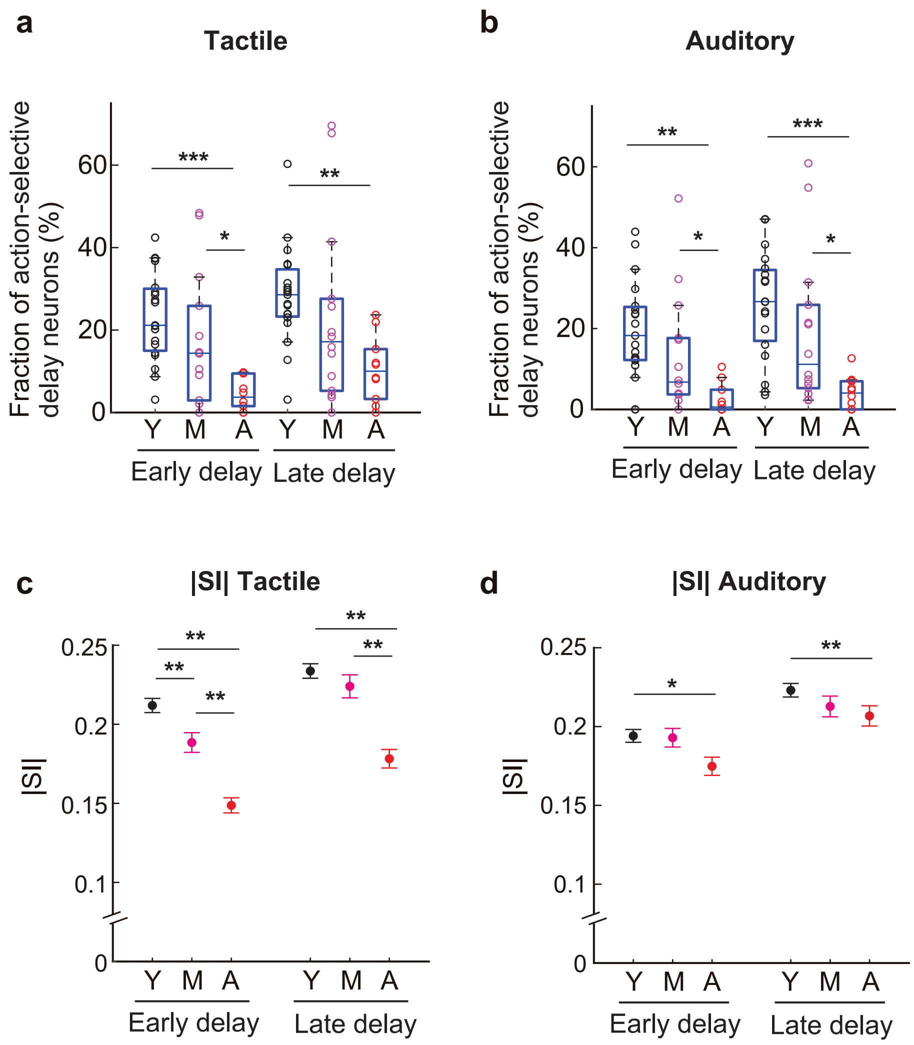

Supplementary Figure 7. Action-plan selective activity during the delay period in the unimodal delayed 2-AFC task. a,b, Fraction of neurons with significant action-plan selective delay activity in the tactile (**a**) and the auditory trials (**b**) for all the imaging sessions (young, *n* = 20 sessions, 10 mice; middle age, *n* = 14 sessions, 7 mice; advanced age, *n* = 10 sessions, 5 mice). Box plots show the median and the 25th and 75th percentiles as box edges and the whiskers extend to the 5th and 95th percentiles. *U =213, p = 0.028, **U =364, p = 3.2 x 10^{-4}, ***U =372, p = 7.2 x 10^{-5} for (**a**), *U =218, p = 0.01 (early delay), *U =219.5, p = 0.01 (late delay), **U =363.5, p = 3.1 x 10^{-4}, ***U =367, p = 1.8 x 10^{-4} for (**b**), Wilcoxon rank-sum test. **c,d,** Mean absolute selectivity index for action plan (|SI|) during the tactile (**c**) and the auditory trials (**d**) (young, *n* = 1,577 neurons; middle age, *n* = 689 neurons; advanced age, *n* = 606 neurons). *p < 0.05, **p < 0.01, one-way ANOVA with Bonferroni post hoc test. Error bars, ± s.e.m. Y, young; M, middle age; A, advanced age. Source data are provided as a Source Data file.

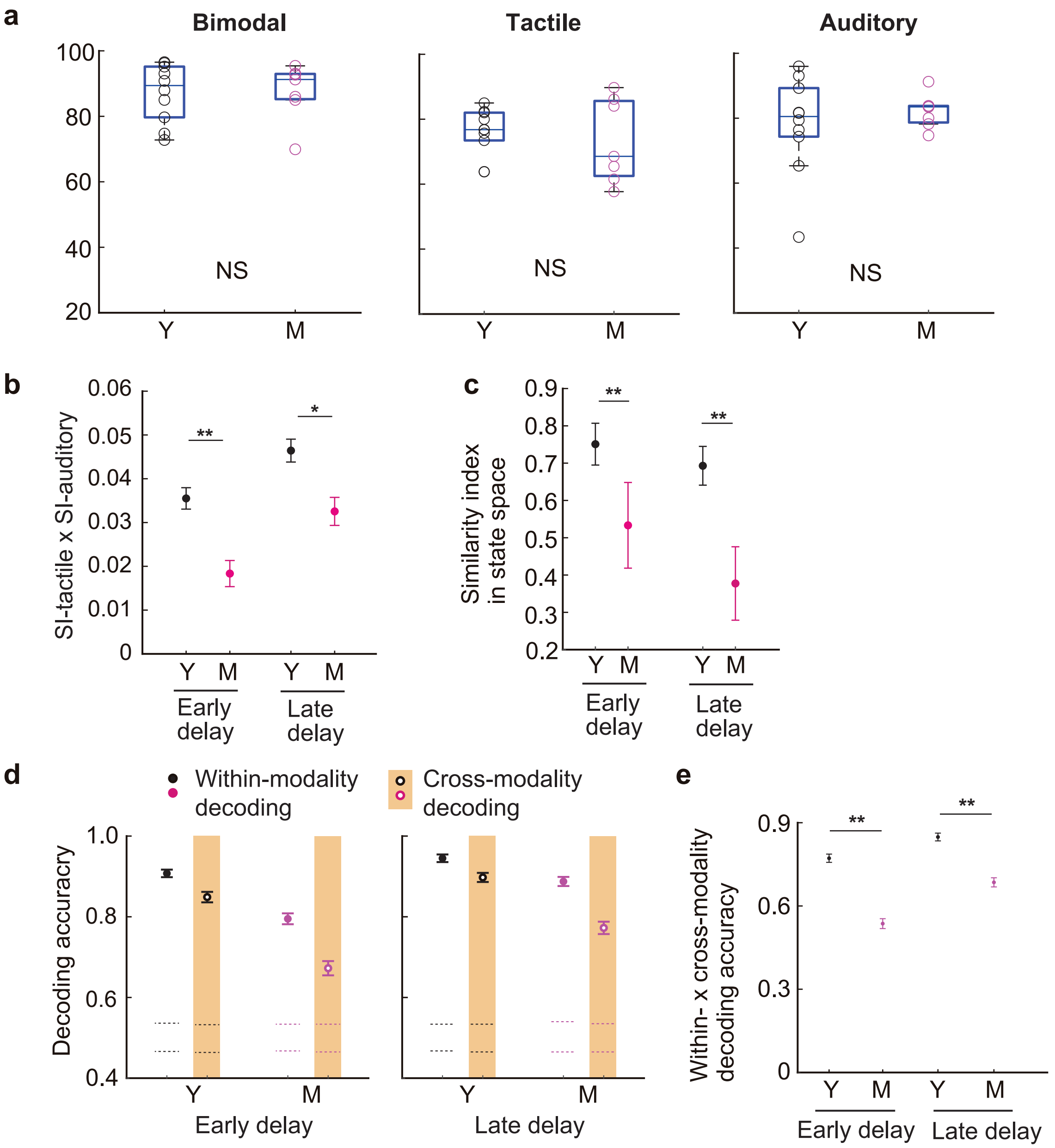

Supplementary Figure 8. Comparisons of modality-general activity using performance-matched middle-aged mice vs. young mice in the auditory task. a, Correct rate in the unimodal d2-AFC task as well as the bimodal task during the imaging sessions (n = 10 young mice, n = 7 middle-aged mice). NS, U = 92, 92, 84, p = 0.89, 0.89, 0.60 for bimodal, tactile, auditory trials, Wilcoxon rank-sum test. Box plots show the median and the 25th and 75th percentiles as box edges and the whiskers extend to the 5th and 95th percentiles. Y, young; M, middle age; A, advanced age. Error bars, ± s.e.m. **b**, Selectivity indices (SIs) in the tactile and the auditory trials were multiplied for each neuron and averaged across all neurons (young, n = 1,577 neurons; middle age, n = 813 neurons). *$F(1,2388)$ = 9.8, $p = 2.0 \times 10^{-3}$, **$F(1,2388)$ = 16.5, $p = 5.0 \times 10^{-5}$, one-way ANOVA. **c**, Similarity index in state space. **$F(1,1998)$ = 3661.2, $p < 1.0 \times 10^{-5}$ for early delay, $F(1,1998)$ = 10396.4, $p < 1.0 \times 10^{-5}$ for late delay, one-way ANOVA. Error bars, ± s.d. across 1,000 iterations. **d**, Decoding accuracy of action plan using within-modality and cross-modality decoding with 200 randomly selected neurons for each age group. The difference in decoding accuracy between within-modality and cross-modality was significantly larger in the middle-aged group compared to the young (W = 1098, p = 0.011 for eary delay and W = 1126, p = 0.016 for late delay; Wilcoxon signed-rank test). Horizontal lines, 97.5th and 2.5th percentile of the shuffled distribution. Error bars, ± s.e.m. across 100 iterations. **e**, Product of multiplying within-modality and cross-modality decoding accuracy. **$F(1,198)$ = 102.9, $p = 9.6 \times 10^{-20}$ for early delay and $F(1,198)$ = 57.4, $p = 1.4 \times 10^{-12}$ for late delay, one-way ANOVA. Error bars, ± s.e.m. across 100 iterations. Source data are provided as a Source Data file.

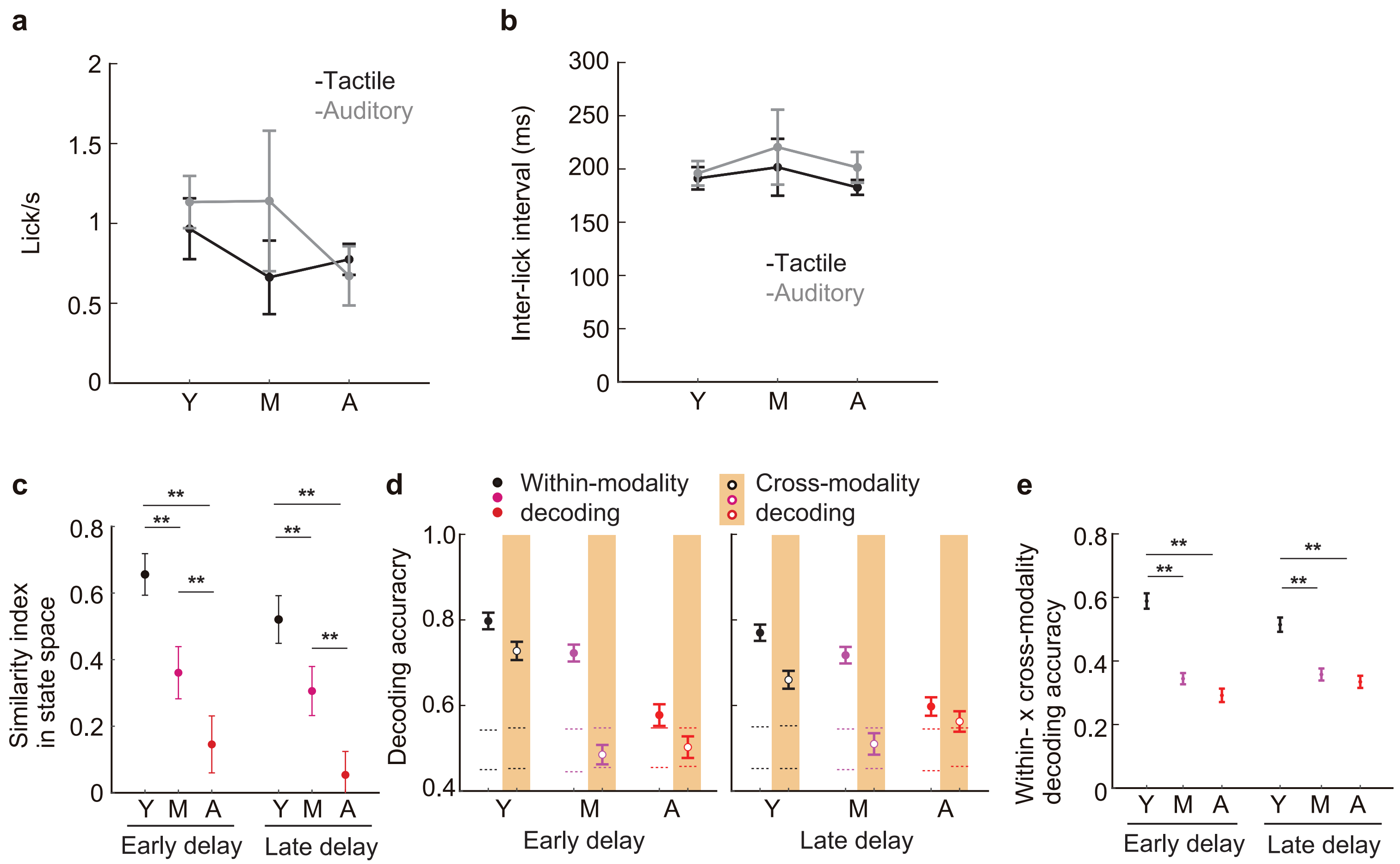

Supplementary Figure 9. Licking response and its effect on modality-general delay activity in the unimodal delayed 2-AFC tasks. a, Mean lick rate during the delay period in the unimodal d2-AFC task averaged across all the mice (*n* = 10 young mice, *n* = 7 middle-aged mice, *n* = 5 advanced-aged mice). The lick rate was not different among the age groups nor between modalities ($F(2,38)$ = 0.94, p = 0.40 for age-group factor, $F(1,38)$ = 0.86, p = 0.36 for modality factor, $F(2,38)$ = 0.66, p = 0.53 for interaction; two-way ANOVA). Error bars, ± s.e.m. Y, young; M, middle age; A, advanced age. **b,** Mean inter-lick interval (ILI) during the delay period. The ILI was not different among the age groups nor between modalities during the delay period ($F(2,37)$ = 0.68, p = 0.51 for age-group factor, $F(1,37)$ = 0.91, p = 0.35 for modality factor, $F(2,37)$ = 0.19, p = 0.83 for interaction; two-way ANOVA). **c-e**, Age-dependent modality-general delay activity in trials with no premature licks during the delay period. **c**, Similarity index in state space. $^{**}p < 0.001$, one-way ANOVA with Bonferroni post hoc test. Error bars, ± s.d. across 1,000 iterations. **d**, Classification accuracy of action plan using within-modality and cross-modality decoding with 200 randomly selected neurons for each age group. Horizontal lines, 97.5th and 2.5th percentile of the shuffled distribution. Error bars, ± s.e.m. across 100 iterations. **e**, Product of multiplying within-modality and cross-modality decoding accuracy. $^{**}p <$ 0.001, one-way ANOVA with Bonferroni post hoc test. Error bars, ± s.e.m. across 100 iterations. Source data are provided as a Source Data file.

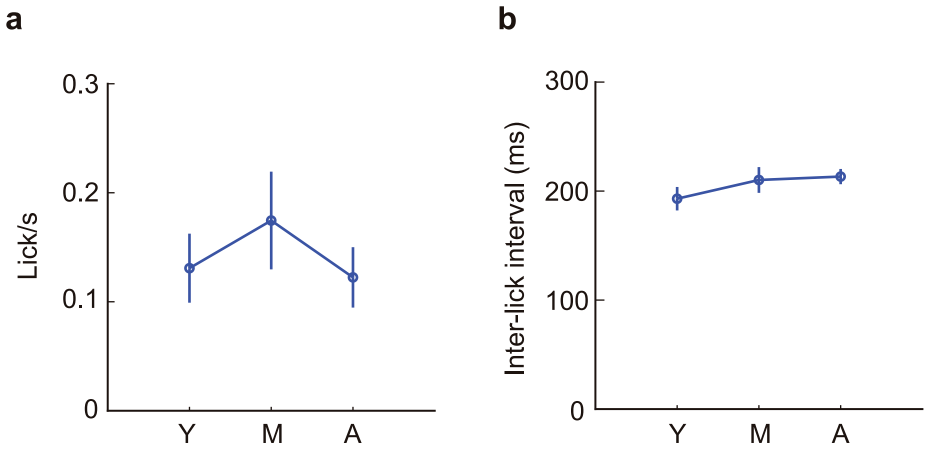

Supplementary Figure 10. Licking response during resting state. a, Mean lick rate did not differ among the age groups during the resting state (*F(2,19)* = 0.61, *p* = 0.56, one-way ANOVA) (young, *n* = 10 mice; middle age, *n* = 7 mice; advanced age, *n* = 5 mice). Error bars, ± s.e.m. **b,** Mean inter-lick interval did not differ among the age groups during the resting state (*F(2,19)* = 1.18, *p* = 0.33, one-way ANOVA). Error bars, ± s.e.m. Source data are provided as a Source Data file.

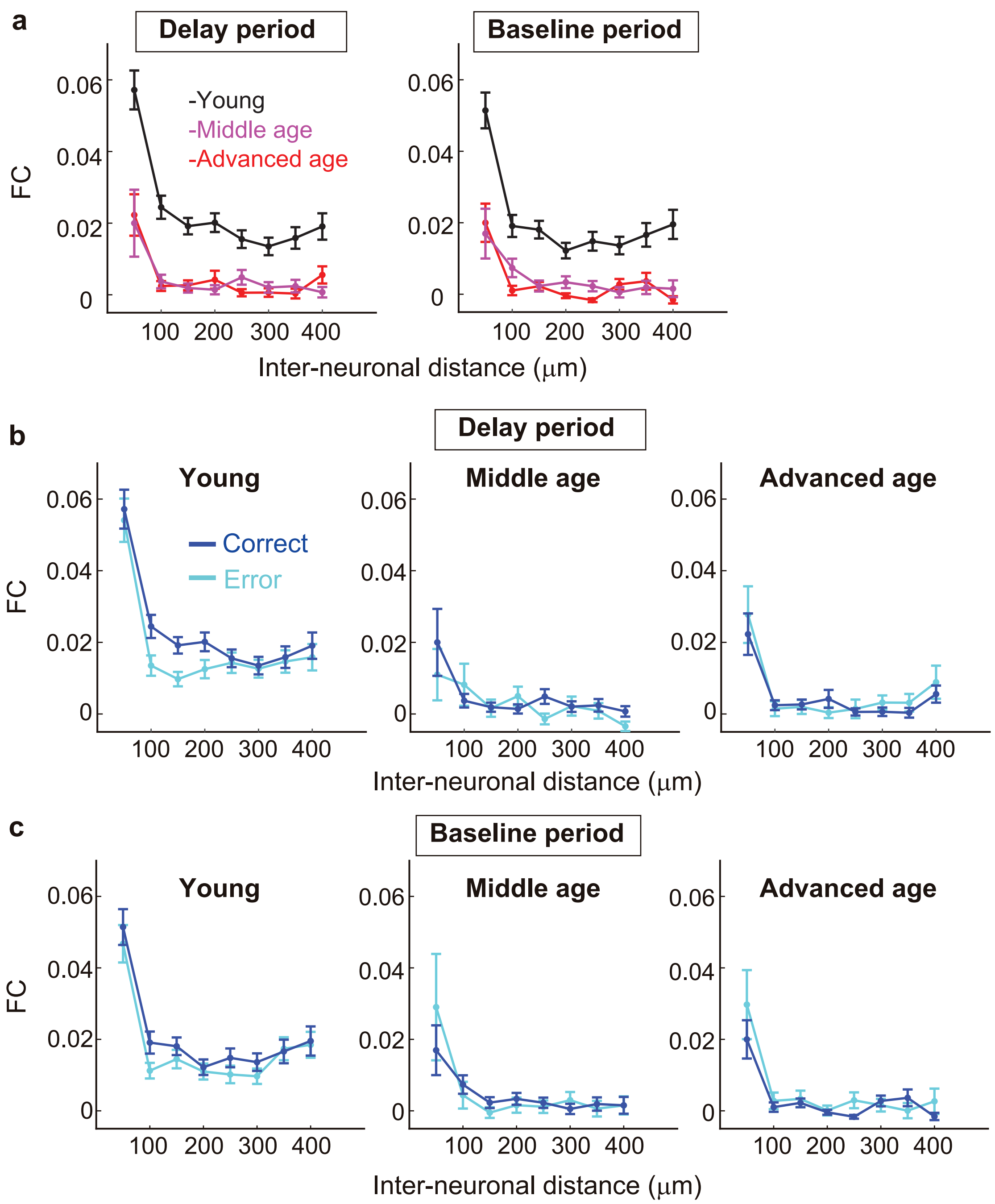

Supplementary Figure 11. Functional connectivity (FC) during the bimodal tasks. a, FC during the delay (left) and baseline (right) periods in the bimodal task as a function of the inter-neuronal distance. **b**, FC in correct trials (blue) and error trials (cyan) was computed between pairs of action-plan-selective delay neurons that had the same sign of action-plan selectivity for each imaging session. Only sessions with 50 or more error trials were included (young, *n* = 6,584 pairs across 441 cells from 9 sessions; middle age, *n* = 1,646 pairs across 175 cells from 7 sessions; advanced age, *n* = 3,925 pairs across 343 cells from 8 sessions). **c**, FC during the baseline period of the same trials and pairs as in b. Error bars denote ± s.e.m. Source data are provided as a Source Data file.

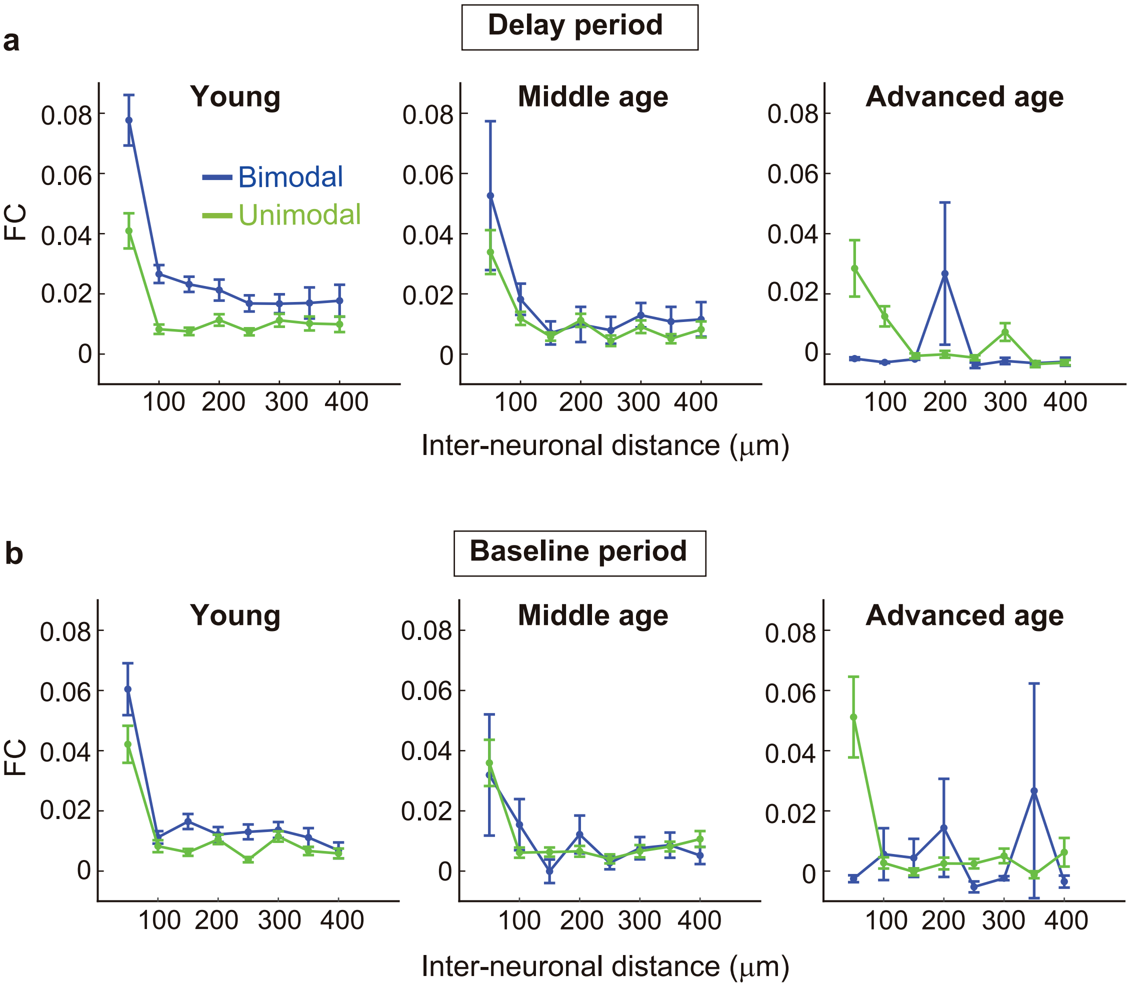

Supplementary Figure 12. Functional connectivity (FC) for bimodal and unimodal neurons. a, FC during the delay period in the unimodal task as a function of the inter-neuronal distance. FC for bimodal neurons (blue) was computed between pairs of action-plan-selective delay neurons that had a significant and the same sign of selectivity index (SI) during the delay period in the tactile and auditory trials (young, *n* = 1,882 pairs across 318 cells from 16 sessions; middle-age, *n* = 266 pairs across 87 cells from 13 sessions; advanced-age, *n* = 75 pairs across 42 cells from 9 sessions). FC for unimodal neurons (green) was computed between pairs of action-plan-selective delay neurons that had a significant SI either in the tactile or auditory trials and did not belong to bimodal neurons (young, *n* = 4,597 pairs across 694 cells; middle-age, *n* = 2,752 pairs across 456 cells; advanced-age, *n* = 1,984 pairs across 348 cells. **b**, FC during the baseline period of the same trials and pairs as in a. Error bars denote ± s.e.m. Source data are provided as a Source Data file.

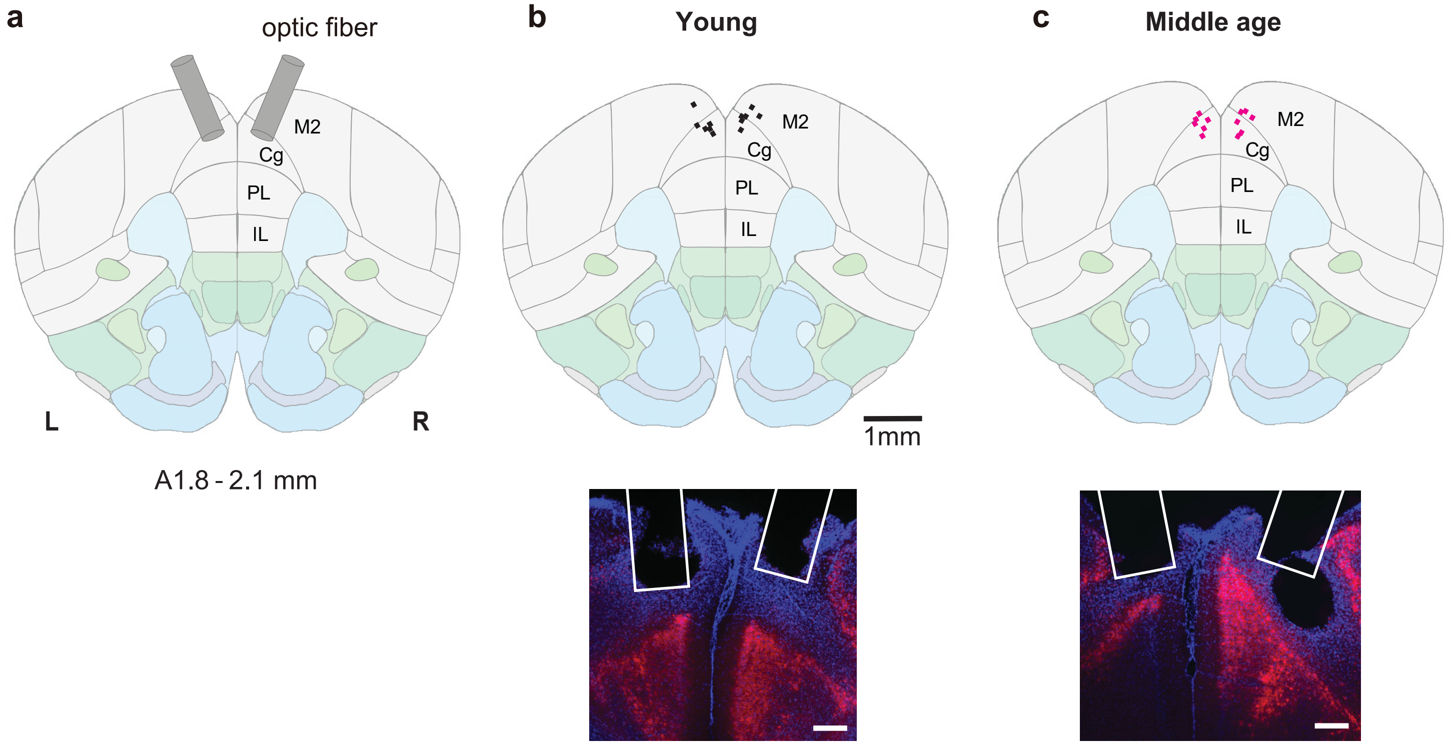

Supplementary Figure 13. Histology for optogenetic inactivation. a, Schematic of bilateral optic fiber implantation. **b,c,** Top, Optic fiber implantation sites in all the CaMKIIα-Cre mice from young (**b**) (*n* = 6 mice) and middle-aged mice (**c**) (*n* = 6 mice), overlaid on the brain atlas modified from Allen Mouse Brain Coronal section (image 38), atlas.brain-map.org. Colored dots denote the tip of optic fibers. Bottom, example images of stGtACR2-FusionRed-expressing neurons (red) stained with DAPI from each age group. White lines, outline of optic fibers. Scale bars, 200 μm. Source data are provided as a Source Data file.

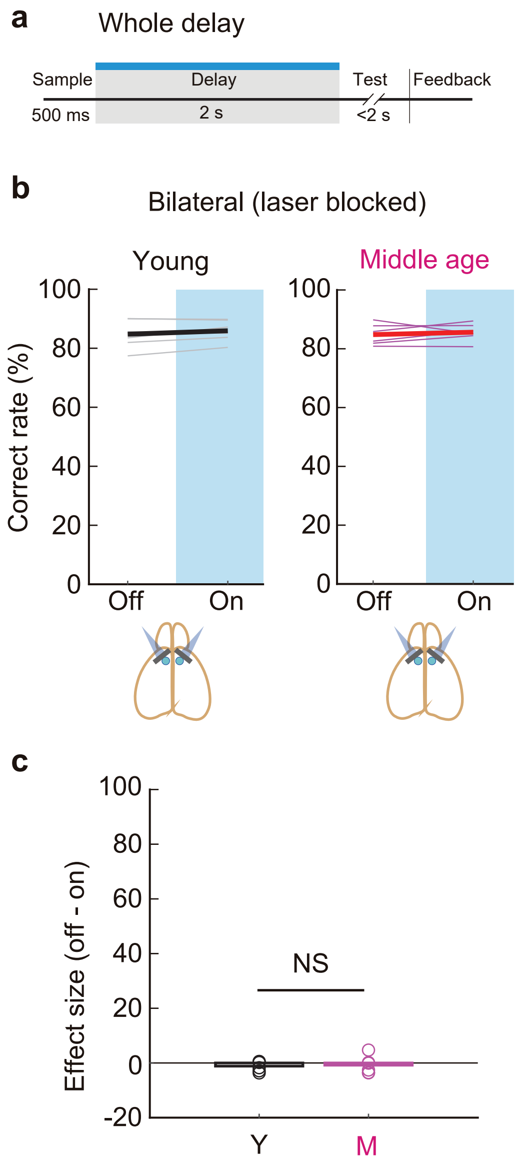

Supplementary Figure 14. Sham optogenetic stimulation. a, Blue laser was delivered during the whole delay period but with blocking the laser pathway to prevent the laser from reaching the brain. **b**, Bilateral sham stimulation did not affect the correct rate either in the young (black lines, n = 6 mice; p = 0.12, one-sided bootstrap; Methods) or the middle-aged mice (magenta lines, n = 6 mice; p = 0.27). Faint colored lines, individual mice. Mouse brain drawings (bottom) modified from Thompson 2020; DOI 10.5281/zenodo.3925942. **c**, Behavioral effect size measured as the difference in the correct rate between laser-off and laser-on conditions. The effect size was not different between the age groups (p = 0.43). Y, young; M, middle age. Source data are provided as a Source Data file.
