# Supplementary material for: Functional alterations of the prefrontal circuit underlying cognitive aging in mice
Source: Nat Commun. 2023 Nov 9;14:7254. doi: 10.1038/s41467-023-43142-0 (PMC10636129; doi:10.1038/s41467-023-43142-0)
Supplement: Supplementary file 3 — Reporting Summary [file 41467_2023_43142_MOESM3_ESM.pdf]

nature portfolio

Corresponding author(s): Tsukasa Kamigaki

Last updated by author(s): Oct 13, 2023

Reporting Summary

Nature Portfolio wishes to improve the reproducibility of the work that we publish. This form provides structure for consistency and transparency in reporting. For further information on Nature Portfolio policies, see our Editorial Policies and the Editorial Policy Checklist.

Statistics

For all statistical analyses, confirm that the following items are present in the figure legend, table legend, main text, or Methods section.

n/a	Confirmed	
☐	☒	The exact sample size (*n*) for each experimental group/condition, given as a discrete number and unit of measurement
☐	☒	A statement on whether measurements were taken from distinct samples or whether the same sample was measured repeatedly
☐	☒	The statistical test(s) used AND whether they are one- or two-sided<br>*Only common tests should be described solely by name; describe more complex techniques in the Methods section.*
☐	☒	A description of all covariates tested
☐	☒	A description of any assumptions or corrections, such as tests of normality and adjustment for multiple comparisons
☐	☒	A full description of the statistical parameters including central tendency (e.g. means) or other basic estimates (e.g. regression coefficient) AND variation (e.g. standard deviation) or associated estimates of uncertainty (e.g. confidence intervals)
☐	☒	For null hypothesis testing, the test statistic (e.g. *F*, *t*, *r*) with confidence intervals, effect sizes, degrees of freedom and *P* value noted<br>*Give P values as exact values whenever suitable.*
☒	☐	For Bayesian analysis, information on the choice of priors and Markov chain Monte Carlo settings
☒	☐	For hierarchical and complex designs, identification of the appropriate level for tests and full reporting of outcomes
☐	☒	Estimates of effect sizes (e.g. Cohen's *d*, Pearson's *r*), indicating how they were calculated

Our web collection on statistics for biologists contains articles on many of the points above.

Software and code

Policy information about availability of computer code

Data collection	Behavior data were acquired with Presentation (23.0) (Neurobehavioral Systems). Calcium imaging data were acquired using Inscopix Data Acquisition Software (IDAS v1.3.0, 1.7.1). Spike data were acquired using Intan RHX Data Acquisition Software (version 3.0.4).
Data analysis	Behavior, imaging, and spike data were analyzed using MATLAB (R2019b, R2022a). Code used to process and analyze data is available on Zenodo (https://zenodo.org/record/8435602). Imaging data were pre-processed using Inscopix Data Processing Software (IDPS v1.8.0). Spike sorting was performed using HerdingSpikes (https://github.com/mhhennig/HS2) and Phy (https://github.com/cortex-lab/phy).

For manuscripts utilizing custom algorithms or software that are central to the research but not yet described in published literature, software must be made available to editors and reviewers. We strongly encourage code deposition in a community repository (e.g. GitHub). See the Nature Portfolio guidelines for submitting code & software for further information.

Data

Policy information about availability of data

All manuscripts must include a data availability statement. This statement should provide the following information, where applicable:

- Accession codes, unique identifiers, or web links for publicly available datasets
- A description of any restrictions on data availability
- For clinical datasets or third party data, please ensure that the statement adheres to our policy

The data generated in this study have been deposited on Zenodo (https://zenodo.org/record/8435602). Source data are provided with this paper.

Human research participants

Policy information about studies involving human research participants and Sex and Gender in Research.

Reporting on sex and gender	N/A
Population characteristics	N/A
Recruitment	N/A
Ethics oversight	N/A

Note that full information on the approval of the study protocol must also be provided in the manuscript.

Field-specific reporting

Please select the one below that is the best fit for your research. If you are not sure, read the appropriate sections before making your selection.

[x] Life sciences [ ] Behavioural & social sciences [ ] Ecological, evolutionary & environmental sciences

For a reference copy of the document with all sections, see nature.com/documents/nr-reporting-summary-flat.pdf

Life sciences study design

All studies must disclose on these points even when the disclosure is negative.

Sample size	Sample sizes were determined according to those reported in previous publications (Kamigaki and Dan, 2017; Pinto and Dan, 2015).
Data exclusions	No data exclusions were conducted.
Replication	All results were replicated across multiple mice in each experiment (n = 11 young, 7 middle-aged, 5 advanced-aged mice for the imaging experiments; n = 6 young, 6 middle-aged for the optogenetic experiments).
Randomization	For all optogenetic stimulation experiments, laser stimulation was applied in 20% of randomly selected trials. Both in the bimodal and unimodal two-alternative forced-choice tasks, left and right trials were randomly interleaved.
Blinding	Investigators could not be blind to age categories as these needed to be identified as per the experimental design. Blinding was not relevant to the data analysis, as identical analysis pipeline and unbiased statistical tests were used.

Reporting for specific materials, systems and methods

We require information from authors about some types of materials, experimental systems and methods used in many studies. Here, indicate whether each material, system or method listed is relevant to your study. If you are not sure if a list item applies to your research, read the appropriate section before selecting a response.

Materials & experimental systems

n/a	Involved in the study	
[x]	[ ]	Antibodies
[x]	[ ]	Eukaryotic cell lines
[x]	[ ]	Palaeontology and archaeology
[ ]	[x]	Animals and other organisms
[x]	[ ]	Clinical data
[x]	[ ]	Dual use research of concern

Methods

n/a	Involved in the study	
[x]	[ ]	ChIP-seq
[x]	[ ]	Flow cytometry
[x]	[ ]	MRI-based neuroimaging

Animals and other research organisms

Policy information about studies involving animals; ARRIVE guidelines recommended for reporting animal research, and Sex and Gender in Research

Laboratory animals	CaMKIIa-Cre mice (Jackson lab stock 005359) were used. Mice were aged 3–8 months (young), 11–14 months (middle-aged), and 18–23 months (advanced-aged). Animals were housed on a 12-h dark/12-h light cycle (light on between 7:00 and 19:00) at around

	21°C and 62% humidity. All the experiments were carried out between 8:00 and 17:00.
Wild animals	No wild animals were used.
Reporting on sex	Both male and female mice were used and described in the manuscript.
Field-collected samples	No field-collected samples were used.
Ethics oversight	All procedures were in accordance with the Institutional Animal Care and Use Committee at Nanyang Technological University.

Note that full information on the approval of the study protocol must also be provided in the manuscript.
